# The aryl hydrocarbon receptor is downregulated in MASLD while its activation attenuates steatosis in human hepatocytes through an IGFBP1-associated pathway

**DOI:** 10.1007/s00204-026-04513-w

**Published:** 2026-07-22

**Authors:** Marta del Pozo-Medina, Polina Soluyanova, Eugenia Pareja, Marta Moreno-Torres, Ramiro Jover

**Affiliations:** 1 CIBEREHD, Instituto de Salud Carlos III, Madrid, Spain; 2 Experimental Hepatology Joint Research Unit. Health Research Institute La Fe, Valencia, Spain; 3 Biochemistry and Molecular Biology Department, Universitat de València, Valencia, Spain; 4 Servicio de Cirugía General y Aparato Digestivo, Hospital Universitario Dr. Peset, Valencia, Spain

**Keywords:** AHR, IGFBP1, MASLD, Steatosis, Lipid accumulation

## Abstract

Metabolic dysfunction–associated steatotic liver disease (MASLD) represents a growing global health concern; however, its multiple pathogenic mechanisms remain incompletely understood. The role of the aryl hydrocarbon receptor (AHR) in MASLD is controversial, as it has been described as either pro- or anti-steatotic depending on the experimental context. Through whole-genome expression profiling and RT-qPCR analysis of human liver biopsies from two independent MASLD patient cohorts, we demonstrate that AHR expression and its canonical activity—assessed by CYP1A1 mRNA levels—are inversely correlated with hepatic steatosis. The role of AHR was further investigated in cultured human Upcyte® hepatocytes from different donors treated with a 2:1 oleate:palmitate mixture (0.6 mM) to induce steatosis, in combination with the exogenous AHR agonist β-naphthoflavone (BN, 25 µM) or the endogenous agonist 2-(1′H-indole-3′-carbonyl)-thiazole-4-carboxylic acid methyl ester (ITE, 25 µM). Activation of AHR resulted in a statistically significant reduction of intracellular lipid levels, as determined by triglyceride quantification and AdipoRed staining. Microarray analysis of AHR-activated human hepatocytes identified IGFBP1 as a robustly induced target gene. Consistently, IGFBP1 expression was diminished in the livers of both MASLD cohorts and positively correlated with AHR expression levels. Furthermore, treatment of human hepatocytes with recombinant IGFBP1, alone or in combination with IGF1, reduced lipid accumulation, supporting IGFBP1 as a potential downstream effector within the anti-steatotic AHR pathway. Collectively, these findings support a role for AHR and its target IGFBP1, as key negative regulators of hepatic lipid accumulation in human hepatocytes and suggest that activation of this pathway may be taken into account in the therapeutic approaches for MASLD.

**Supplementary Information:**

The online version contains supplementary material available at https://doi.org/10.1007/s00204-026-04513-w.

## Introduction

Metabolic dysfunction-associated steatotic liver disease (MASLD) is the most common chronic liver disease worldwide and is becoming a leading indication for liver-related morbidity and transplantation. It comprises a disease spectrum ranging from simple steatosis to steatohepatitis, fibrosis, cirrhosis, and hepatocellular carcinoma, and is closely linked to obesity, insulin resistance, type 2 diabetes, gut microbial dysbiosis, and genetic susceptibility (Le et al. 2025; Huang et al. 2025). Although progression to advanced disease stages determines the long-term clinical outcome, hepatic triglyceride accumulation is the defining hallmark of MASLD. In addition, inflammatory, oxidative, and endocrine signals, including those related to the insulin/IGF axis, further shape lipid handling and disease progression (Marigota et al. 2024). Therefore, identifying pathways that restrain lipid accumulation in human hepatocytes remains an important objective in MASLD research.

The aryl hydrocarbon receptor (AHR) is a ligand-activated transcription factor of the basic helix-loop-helix/Per-Arnt-Sim family that was initially characterized as a mediator of xenobiotic toxicity induced by environmental contaminants such as dioxins and polycyclic aromatic hydrocarbons (Kolluri et al. 2017). Upon ligand binding, AHR translocates to the nucleus, dimerizes with ARNT, and binds xenobiotic response elements to regulate canonical target genes involved in xenobiotic metabolism/bioactivation, including enzymes such as CYP1A1 (Walisser et al. 2005). Beyond this classical pathway, AHR is now recognized as a broader environmental and metabolic sensor capable of integrating exogenous ligands with endogenous and microbiota-derived metabolites to modulate inflammation, cell fate, metabolic adaptation, and tissue homeostasis (Wright et al. 2017; Patil et al. 2023).

The role of AHR in hepatic steatosis and MASLD remains controversial. In several experimental settings, particularly under sustained exposure to high-affinity xenobiotic ligands, AHR activation has been associated with hepatic lipid accumulation, oxidative stress, and metabolic dysfunction. This experimental evidence led to the proposal of AHR as a Molecular Initiating Event (MIE) in the Adverse Outcome Pathway (AOP) of toxic steatosis (Mellor et al. 2016; Zheng et al. 2024), implying that any chemical activating AHR could be labelled as potentially steatotic.

In contrast, other studies have shown that hepatocyte AHR deficiency may aggravate diet-induced steatosis and liver injury, and that activation of AHR by selected endogenous or microbiota-derived ligands can exert hepatoprotective effects by limiting lipogenesis and inflammatory signaling (Wada et al. 2016; Patil et al. 2023).

These apparently opposite findings support a context-dependent model in which the metabolic consequences of AHR signaling depend on ligand identity, exposure intensity and duration, tissue specificity, and downstream transcriptional programs. However, despite the growing interest in AHR as a metabolic regulator, evidence directly addressing its relationship with steatosis in the human liver is still limited.

In this study, we investigated whether AHR is associated with a pro- or anti-steatotic phenotype in humans by combining human liver tissue analyses with functional studies in cultured human hepatocytes. Specifically, we evaluated hepatic AHR expression and canonical AHR activity, assessed by CYP1A1 expression, in two independent human cohorts spanning a wide range of steatosis. We then examined whether AHR activation modifies lipid accumulation in fatty acid-loaded human hepatocytes and explored downstream genes induced by AHR that could contribute to this phenotype. Using this translational approach, we identified a negative association between the AHR pathway and hepatic steatosis in the human liver, highlighting IGFBP1 as a candidate effector of the AHR-induced anti-steatotic response.

## Material and methods

### Human samples and baseline characteristics of patients

Liver tissue samples were obtained from two independent cohorts. The first cohort included 33 patients, from whom scheduled wedge liver biopsies were collected intraoperatively at the time of bariatric surgery (Pérez-Rubio et al. 2023) (Table 1). These morbidly obese patients, who were referred to the Esophageal-Gastric Surgery Section of Dr. Peset University Hospital in Valencia, were recruited between 2020 and 2023. All participants met the 1991 NIH criteria for bariatric surgery (1992). Exclusion criteria included obesity secondary to endocrinopathies, active malignancy, severe psychiatric disorders, alcoholism or substance dependence, non-compliance with recommended dietary and lifestyle modifications, anesthetic contraindications, and serious comorbidities. From this cohort a subset of 10 patients was selected for whole transcriptome microarray analyses.

**Table 1:** Baseline characteristics of the bariatric surgery study population.

	Mean/%	SD	Median	IQR	Max	Min
*Demographic-related data*						
Gender Female	70%					
Age (years)	47	12	48	21	63	20
BMI (kg/m^2^)	38	4	38	6	51	31
*Pre-existing conditions*						
T2DM	36%					
High blood pressure	52%					
Hypercholesterolemia	55%					
Hypertriglyceridemia	33%					
*Biochemistry*						
Glucose (mg/dL)	101	19	95	17	172	78
HbA1c (%)	5.7	0.7	5.5	0.9	7.9	4.6
Insulin (µU/mL)	16.0	6.1	16.6	11.1	31.8	7.4
HOMA-IR	4.0	1.6	4.4	2.4	7.3	1.4
Cholesterol (mg/dL)	169	46	167	56	280	70
HDL (mg/dL)	42	10	42	13	65	26
LDL (mg/dL)	99	43	95	43	199	10
Triglycerides (mg/dL)	143	61	137	90	304	67
Bilirubin (mg/dL)	0.64	0.52	0.49	0.40	2.48	0.25
AST (IU/L)	18	5	17	6	38	9
ALT (IU/L)	21	11	17	12	57	9
GGT (IU/L)	23	12	20	9	67	8
ALP (IU/L)	76	22	70	23	138	38
Albumin (g/dL)	4.3	0.3	4.3	0.4	4.8	3.6
PT (s)	11	1	11	1	13	10
INR	1.0	0.1	1.0	0.1	1.1	0.9
Hemoglobin (g/dL)	14	1	14	2	17	12
Platelets (10^3/µL)	260	63	247	79	378	112
*Histopathology & imaging*						
% Fat (digital image)	3.11	3.78	1.81	2.82	15.22	0.16
MRI-PDFF (Dixon)	10.1	6.1	8.9	7.1	29.8	2.3
IR (Lipid/Prot)	0.067	0.054	0.045	0.061	0.214	0.013
*Steatosis grade (% of patients)*						
	**0**	**1**	**2**	**3**	**4**	
Steatosis	15%	64%	21%	0%		
Ballooning	53%	38%	9%			
Lob Inflammation	31%	66%	3%			
Fibrosis	70%	27%	0%	0%	3%	

The second cohort consisted of 44 liver samples from deceased donors, obtained from La Fe Hospital Liver Biobank (Valencia, Spain) (Table 2). This cohort was described previously (Quintás et al. 2022) and involved organ donors who were initially designated for liver transplantation, but whose livers could not be transplanted and were instead donated for research purposes. Triglyceride (TG) levels were measured using biochemical analysis.

**Table 2:** Baseline characteristics of the Liver Biobank study population.

	Mean /%	SD	Median	IQR	Max	Min
*Demographic-related data*						
Gender Female	41%					
Age (years)	55	18	60	20	80	15
BMI (kg/m^2^)	28	4	27	6	34	21
*Pre-existing conditions*						
Cause of death: Stroke/TBI	76%					
Clinical history: HBP/T2DM/DYSLIP	41%					
*Biochemistry*						
Glucose (mg/dL)	187	58	179	56	369	99
Urea (mg/dL)	38	17	38	25	77	14
Creatinine (mg/dL)	2.0	2.8	0.9	0.5	12.0	0.5
Cholesterol (mg/dL)	147	83	171	147	218	28
Triglycerides (mg/dL)	115	64	122	124	182	35
Bilirubin (mg/dL)	3.4	10.4	0.8	1.1	55.0	0.1
AST (IU/L)	67	72	36	51	342	15
ALT (IU/L)	66	94	35	46	355	13
ALP (IU/L)	103	62	92	63	228	23
GGT (IU/L)	58	68	26	62	255	10
LDH (IU/L)	509	336	446	357	1473	163
CK (IU/L)	598	1058	119	698	3859	14
Proteins (g/dL)	11	17	5	2	64	2
Fibrinogen (mg/dL)	368	195	344	209	894	5
PT (s)	67	24	71	33	103	9
APTT/aPTT (s)	33	10	31	6	64	24
Hemoglobin (g/dL)	17	24	11	2	112	4
Platelets (10^3^/µL)	231	150	187	205	537	69
*Steatosis-related data*						
Macro-Steatosis (%)	41	25	38	46	80	5
Tot. Lipids (µg/mg prot)	587	622	292	741	2704	7
Triglycerides (µg/mg prot)	422	454	226	557	1944	25

TBI: traumatic brain injury

HBP/T2DM/DYSLIP: High Blood Pressure/Type 2 Diabetes Mellitus/Dyslipidemia

Both studies were conducted in accordance with the Declaration of Helsinki, were approved by the Human Ethics Committees of La Fe University Hospital (n° 2013/0232, 2012/0452 and 2022–204-1) and Dr. Peset University Hospital (n° 44/19) in Valencia, Spain, and written informed consent was obtained from all patients prior to participation.

Both cohorts displayed variable hepatic fat content, ranging from individuals without steatosis to MASLD patients with significant steatosis, as assessed by an experienced pathologist according to standard histopathological criteria, as well as by additional quantitative methods (digital biopsy image quantification, MRI, infrared spectroscopy (IR), and biochemical lipid/triglyceride quantification) (Quintás et al. 2022; Pérez-Rubio et al. 2023; Soluyanova et al. 2024).

Clinical and biochemical baseline data were recorded for each cohort, including age, body mass index (BMI), fasting glucose, lipid profile, and liver function tests.

### Cell culture and treatments

Upcyte hepatocytes are derived from primary human hepatocytes using a proprietary technology that enables controlled proliferation without immortalization. Unlike conventional hepatic cell lines, these cells retain gene expression profiles and functional properties that closely resemble those of cultured primary human hepatocytes (Levy et al. 2015; Tolosa et al. 2016). Cells and culture media were obtained from Upcyte Technologies (ClinicSciences Group). Upcyte® human hepatocytes from multiple donors were used to enhance the robustness and translational relevance of the study.

Cells were initially seeded in collagen-coated Corning T75 flasks (75 cm^2^) at a density of 3.5 × 10^5 cells per flask and maintained at 37 °C in a humidified atmosphere containing 5% CO₂. Culture medium was replaced every 48 h. At approximately 75% confluence, cells were detached using trypsin and reseeded onto collagen-coated plates at densities of 2.0 × 10^5 cells/well in 24-well plates and 2.0 × 10^4 cells/well in 96-well plates. After 4–5 h, the medium was replaced with a high-performance medium, and cells were allowed to differentiate for 3–4 days as previously described (Tolosa et al. 2016), with medium changes every 48 h.

To induce steatosis, differentiated hepatocytes were pretreated with 0.6 mM oleate:palmitate (2:1) for 24 h. In parallel, cells were exposed for 24 h to the AHR activators β-naphthoflavone (BN, 25 µM; CAS 6051–87-2; Thermo Fisher Scientific, A18543.06) and 2-(1′H-indole-3′-carbonyl)-thiazole-4-carboxylic acid methyl ester (ITE, 25 µM; CAS 448906–42-1; Sigma-Aldrich, SML3139), using DMSO as the solvent at a final concentration of 0.05%, in order to evaluate the effect of AHR activation on lipid accumulation. Recombinant human proteins IGF-1 and IGFBP-1 used in this study were obtained from Thermo Fisher Scientific (PeproTech, catalog nos. 100–11-500UG and 350–10-25UG, respectively) and were used either individually or in combination at a concentration of 150 ng/mL for each protein.

### RNA purification and cDNA synthesis

Total RNA was isolated from hepatocytes cultured in 24-well plates using the E.Z.N.A. Total RNA Kit (Omega Bio-tek, R6731-01) according to the manufacturer’s instructions. Cells were lysed directly in the culture wells to maximize RNA yield and preserve RNA integrity. RNA concentration and purity were determined using a NanoPhotometer→N60 microvolume spectrophotometer (Implen GmbH, Munich, Germany), and samples were stored at -80 °C until further analysis.

Complementary DNA (cDNA) was synthesized from 1 µg of total RNA using Moloney Murine Leukemia Virus (M-MLV) reverse transcriptase (Invitrogen™, Thermo Fisher Scientific, Waltham, MA, USA) following the manufacturer’s protocol.

### Microarray analysis

RNA integrity was evaluated by microcapillary electrophoresis using the 2100 Bioanalyzer (Agilent Technologies), and quality was assessed based on the RNA Integrity Number (RIN). Global transcriptomic profiling was performed using Clariom S microarrays (Thermo Fisher Scientific). RNA processing, microarray hybridization, and slide scanning were carried out at the Central Unit for Research in Medicine (UCIM), University of Valencia. Quality control and raw data acquisition were performed using the Transcriptome Analysis Console (Applied Biosystems).

Two independent analyses, with the same workflow, were performed: one with liver RNA samples from 10 bariatric surgery patients, including 5 with MASLD and 5 without MASLD; and another with RNA samples from Upcyte® human hepatocytes treated with either DMSO (n = 4) or the AHR agonist BN (n = 4). Microarray data were analyzed using Transcriptome Analysis Console (TAC) software (Thermo Fisher Scientific). Only genes detected in both conditions (non-MASLD vs. MASLD in patient samples; or DMSO vs. BN in hepatocytes) were retained.

Pathway enrichment analysis of differentially expressed genes was performed using ConsensusPathDB (Max Planck Institute for Molecular Genetics), applying a significance threshold of *p* < 0.01.

Microarray data have been deposited in the Zenodo repository under https://doi.org/10.5281/zenodo.20079491.

### RT-qPCR

Complementary DNA (cDNA) was diluted 1:30 in TE buffer supplemented with quinoline (1:200) and used as template for quantitative real-time PCR (RT–qPCR) analyses. RT–qPCR was performed to determine the expression of selected genes and to validate candidate genes identified by transcriptomic microarray analysis.

Gene-specific primers were purchased from Integrated DNA Technologies (Supplementary Table 1). Amplification reactions were performed under standard conditions using a LightCycler 480 II real-time PCR system (Roche). Fluorescence was monitored in real time to obtain cycle threshold (Ct) values and relative expression levels.

Gene expression was normalized using the housekeeping genes PBGD and RPLP0. In addition, a reference calibrator cDNA, generated from a pool of 20 human liver samples (La Fe Hospital Liver Biobank, Valencia, Spain), was included in each run. Besides biological replicates, all reactions were performed in technical duplicates.

### Steatosis quantification in liver biopsies and cultured hepatocytes

In the cohort of obese patients, hepatic lipid content was initially evaluated using several complementary quantitative methods, including digital image analysis of hematoxylin and eosin (H&E)-stained liver biopsy sections, MRI-derived proton density fat fraction (MRI-PDFF), and infrared spectroscopy (IR), as previously described (Quintás et al. 2022; Pérez-Rubio et al. 2023; Soluyanova et al. 2024). Among these, steatosis percentage determined by digital image analysis and MRI-PDFF were selected as the primary outcome measures because of their higher precision and broad clinical acceptance.

In the liver biobank cohort, TG content was measured by methanol–chloroform lipid extraction of homogenized liver biopsies, and solvent evaporation in a SpeedVac, followed by TG quantification using a commercial enzymatic kit (Spinreact, Barcelona, Spain). TG levels were normalized to total protein content.

In Upcyte® hepatocytes, intracellular lipid accumulation was assessed using two complementary methods. First, intracellular TGs were extracted and quantified with a commercial kit as described above. Second, neutral lipid content was measured using the AdipoRed™ assay (Lonza), which enables direct intracellular lipid quantification without prior extraction. For this assay, cells seeded in opaque 96 well plates, treated with the corresponding treatment and afterwards medium was replaced with new medium with AdipoRed (5 L/ 200 L medium) and Hoechst 33342 (1 g/m L) for 30 min at 37 °C, and fluorescence was recorded using a Synergy H1 multimode microplate reader (BioTek/Agilent Technologies, Wilmington, DE, USA). Lipid-associated fluorescence was normalized to cell number as determined by Hoechst staining or by protein content.

### Data analysis and statistics

Quantitative variables are presented as mean ± standard deviation (SD), median, interquartile range (IQR), minimum, and maximum, as appropriate. Categorical variables are presented as number and percentage. For continuous variables reported in Tables 1 and 2, the IQR was calculated as the difference between the third quartile and the first quartile: IQR = Q3-Q1, where Q1 represents the 25th percentile and Q3 the 75th percentile. Biological replicates (*n*) consisted of independent experiments performed sequentially, each including 2–3 technical replicates. Comparisons between two groups were carried out using Student’s *t* test. Correlations between quantitative variables were assessed using Pearson’s correlation coefficient (r). A *p* value < 0.05 was considered statistically significant. Statistical analyses were performed using GraphPad Prism.

## Results

### Hepatic steatosis is negatively associated with the AHR pathway in human liver

#### Microarray analyses reveals downregulation of the AHR pathway in obese patients with MASLD

Microarray analysis was performed to identify genes that were upregulated and downregulated in liver biopsies from 10 selected bariatric-surgery obese patients with high (n = 5) and low (n = 5) liver fat content. In this small subgroup of patients, the % of liver fat, measured by digital biopsy imaging, was 3.9-fold higher in the high-fat group than in the low-fat group (*p* = 0.0010). Similar results were observed when the liver lipid content was measured by MRI-PDFF (3.7-fold higher, *p* = 0.0045).

**Fig. 1:**
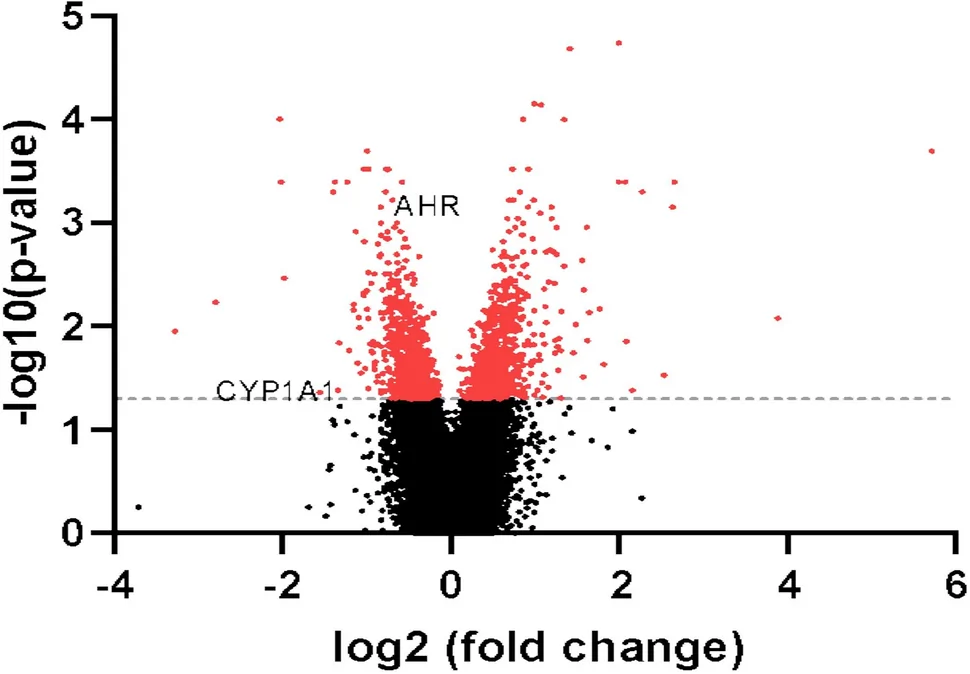
Volcano plot depicting differentially expressed genes in obese MASLD patients (n = 5) compared with obese non-MASLD patients (n = 5). Each dot represents one gene, displayed according to its log2 fold change (x-axis) and -log10 (*p*-value) (y-axis). Significant upregulated and downregulated genes (± 1.6-fold change, *p* < 0.05) are highlighted in red.

Comparative gene expression analysis between the high and low liver fat groups identified a total of 310 genes significantly deregulated in association with steatosis (± 1.6-fold change, p < 0.05) (Supplementary Table 2). Among these, 186 genes were positively associated with hepatic lipid accumulation, whereas 124 showed a negative association. The top 25 upregulated and downregulated genes are presented in Table 3, and the corresponding volcano plot is shown in Fig. 1.

**Table 3:** Top 25 upregulated and downregulated genes in high versus low fat liver samples from obese MASLD patients as ranked by fold-change.

Gene	Fold change	*P*-value	FDR *P*-value	Description
IGFBP1	-9.76	0.0111	0.6725	Insulin like growth factor binding protein 1
IGFBP2	-7.03	0.0058	0.6494	Insulin like growth factor binding protein 2
SHBG	-4.13	9.84E-05	0.3926	Sex hormone-binding globulin
P4HA1	-4.09	0.0004	0.4382	Prolyl 4-hydroxylase, alpha polypeptide I
GREB1	-3.99	0.0034	0.6494	Growth regulation by estrogen in breast cancer 1
CYP1A1	-2.95	0.0434	0.7351	Cytochrome P450, family 1, subfamily A, polypeptide 1
SOCS2	-2.66	0.0005	0.4382	Suppressor of cytokine signaling 2
CISH	-2.62	0.0004	0.4382	Cytokine inducible SH2-containing protein
FST	-2.56	0.0412	0.7351	Follistatin
SAT2	-2.52	0.0143	0.7048	Spermidine/spermine N1-acetyltransferase family member 2
WNK3	-2.35	0.0004	0.4382	WNK lysine deficient protein kinase 3
CYP3A43	-2.33	0.017	0.7163	Cytochrome P450, family 3, subfamily A, polypeptide 43
PTP4A3	-2.25	0.0061	0.6494	Protein tyrosine phosphatase type IVA, member 3
NSA2	-2.24	0.0069	0.6494	NSA2 ribosome biogenesis homolog
SLC16A1	-2.2	0.0012	0.5249	Solute carrier family 16 (monocarboxylate transporter), member 1
NEK10	-2.18	0.0226	0.7253	NIMA-related kinase 10
ADAMTSL1	-2.16	0.0081	0.6535	ADAMTS like 1
CNDP1	-2.14	0.0102	0.6589	Carnosine dipeptidase 1 (metallopeptidase M20 family)
CUX2	-2.12	0.028	0.7288	Cut-like homeobox 2
FOXP1	-2.08	0.0003	0.4382	Forkhead box P1
DNAJC12	-2.07	0.005	0.6494	DnaJ (Hsp40) homolog, subfamily C, member 12
CHML	-2.06	0.0015	0.5382	Choroideremia-like (Rab escort protein 2)
ZCCHC6	-2.06	0.0047	0.6494	Zinc finger, CCHC domain containing 6
SLC16A1	-2.04	0.0252	0.7253	Solute carrier family 16 (monocarboxylate transporter), member 1
RPL36A	-2.02	0.0068	0.6494	Ribosomal protein L36a
ENO3	2.46	0.0071	0.6494	Enolase 3 (beta, muscle)
CDHR5	2.51	0.0026	0.6234	Cadherin-related family member 5
FAT1	2.52	0.0001	0.3926	FAT atypical cadherin 1
HSPA4L	2.64	2.07E-05	0.222	Heat shock 70 kDa protein 4-like
CXCL10	2.71	0.0178	0.7163	Chemokine (C-X-C motif) ligand 10
PDE11A	2.77	0.0096	0.6589	Phosphodiesterase 11A
IL32	2.91	0.0023	0.6234	Interleukin 32
HSPA1B; HSPA1A	2.96	0.0305	0.7313	Heat shock 70 kDa protein 1B; heat shock 70 kDa protein 1A
IDI1	2.98	0.0044	0.6494	Isopentenyl-diphosphate delta isomerase 1
SULF2	3.02	0.0011	0.5249	Sulfatase 2
ELOVL6	3.05	0.0136	0.6938	ELOVL fatty acid elongase 6
TMEM45B	3.1	0.0073	0.6494	Transmembrane protein 45B
GSTM3	3.38	0.0067	0.6494	Glutathione S-transferase mu 3 (brain)
FASN	3.48	0.0232	0.7253	Fatty acid synthase
PPP1R3B	3.94	0.0004	0.4382	Protein phosphatase 1, regulatory subunit 3B
TMEM154	3.95	1.81E-05	0.222	Transmembrane protein 154
GSTM4	4.17	0.0004	0.4382	Glutathione S-transferase mu 4
SAA2; SAA4	4.19	0.0139	0.6938	Serum amyloid A2; serum amyloid A4
LGALS4	4.41	0.0413	0.7351	Lectin, galactoside-binding, soluble, 4
GSTM2	4.79	0.0005	0.4382	Glutathione S-transferase mu 2 (muscle)
HMGCS1	5.73	0.0294	0.7313	3-hydroxy-3-methylglutaryl-CoA synthase 1 (soluble)
GSTM5	6.12	0.0007	0.4424	Glutathione S-transferase mu 5
TBX15	6.22	0.0004	0.4382	T-box 15
MUC3A	14.64	0.0083	0.6535	Mucin 3A, cell surface associated
GSTM1	52.01	0.0002	0.4382	Glutathione S-transferase mu 1

The fold changes, calculated as the ratios of gene expression between the subjects with high- and low-fat content, varied between + 52.0 (GSTM1) and -9.8 (IGFBP1). The genes significantly upregulated in steatosis covered a wide range of biological functions and could be annotated in 23 overrepresented metabolic pathways (*p* < 0.001; Sources: Reactome, Wiki pathways and KEGG), many of them related to cholesterol/sterol metabolism, as well as lipid glutathione metabolism and xenobiotic biotransformation. Regarding genes downregulated in steatosis, they belonged to pathways such as arginine and proline metabolism, HIF-1 survival signaling and AHR Pathway (*p* < 0.005) (Supplementary Table 3A). Regarding this last pathway, AHR and its bona fide target gene CYP1A1 were significantly repressed in steatosis -1.8- and -3.0- fold, respectively (Supplementary Table 2).

#### RT-qPCR analysis confirms the negative correlation of AHR and CYP1A1 expression with liver fat content

Given the limited sample size of the microarray analysis (n = 10), the obese cohort was expanded to 33 patients. Rather than categorizing patients into low- and high-fat groups, we took advantage of the precise quantification of hepatic steatosis as a continuous variable using multiple methods (% of liver fat from digital images and MRI-PDFF) and performed Pearson correlation analyses.

**Fig. 2:**
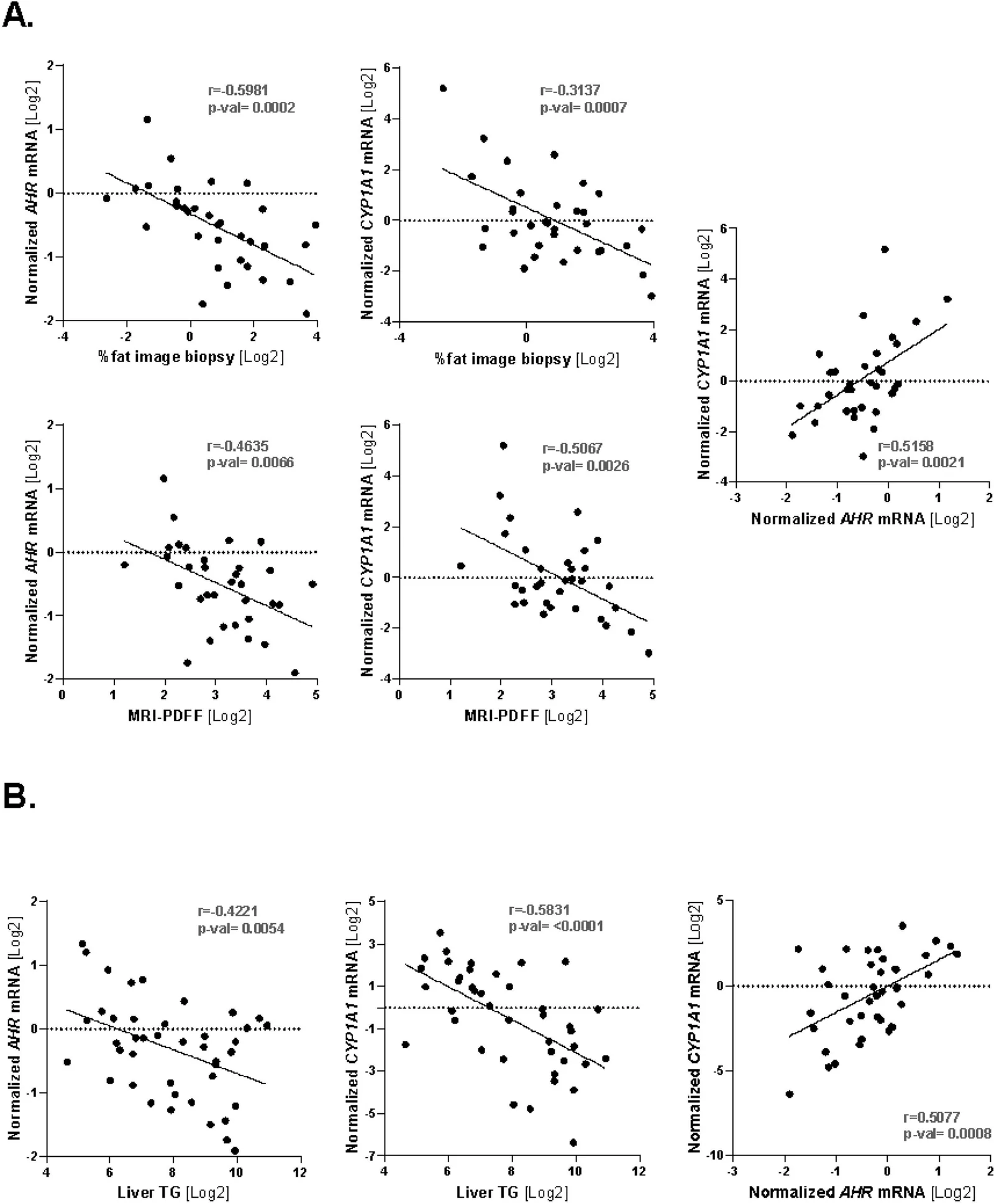
Correlation analyses of AHR and CYP1A1 expression levels with hepatic steatosis parameters. **A** Correlations of AHR and CYP1A1 mRNAs with % of liver fat and MRI-PDFF values in obese patients undergoing bariatric surgery. A strong positive correlation between AHR and CYP1A1 expression is also shown (right). **B** Correlations in the non-obese liver biobank cohort, demonstrating again negative associations between AHR / CYP1A1 mRNAs and hepatic TG levels, as well as a positive correlation between AHR and CYP1A1 expression (right). r denotes Pearson’s correlation coefficient, measuring the strength and direction of the linear relationship between variables. The *p*-value (*p*-val) indicates the statistical significance of the correlation, testing whether the observed association differs from zero.

Focusing on the AHR/CYP1A1 pathway, the results confirmed a significant inverse correlation between hepatic steatosis and the liver mRNA levels of these two genes. Moreover, AHR and CYP1A1 expression strongly and positively correlated with each other (Fig. 2A).

To further validate these findings and rule out obesity-related factors behind gene dysregulation, a second cohort of non-obese patients (n = 44), spanning a wide range of steatosis, was analyzed by RT-qPCR. The same pattern of inverse correlation between steatosis (measured as TG content) and AHR/CYP1A1 was observed, reinforcing the negative association of these genes with hepatic fat accumulation independently of obesity (Fig. 2B).

### AHR activation reduces lipid accumulation in cultured human hepatocytes

The downregulation of AHR in steatotic livers may represent either a compensatory or a pathogenic mechanism. To investigate its role, we used a human in vitro model of hepatic steatosis. Human hepatocytes were treated with a mixture of fatty acids (oleate:palmitate, 2:1 ratio, 0.6 mM) in combination with either the solvent DMSO or AHR activators: BN (a canonical AHR agonist) and ITE (a more potent AHR activator).

**Fig. 3:**
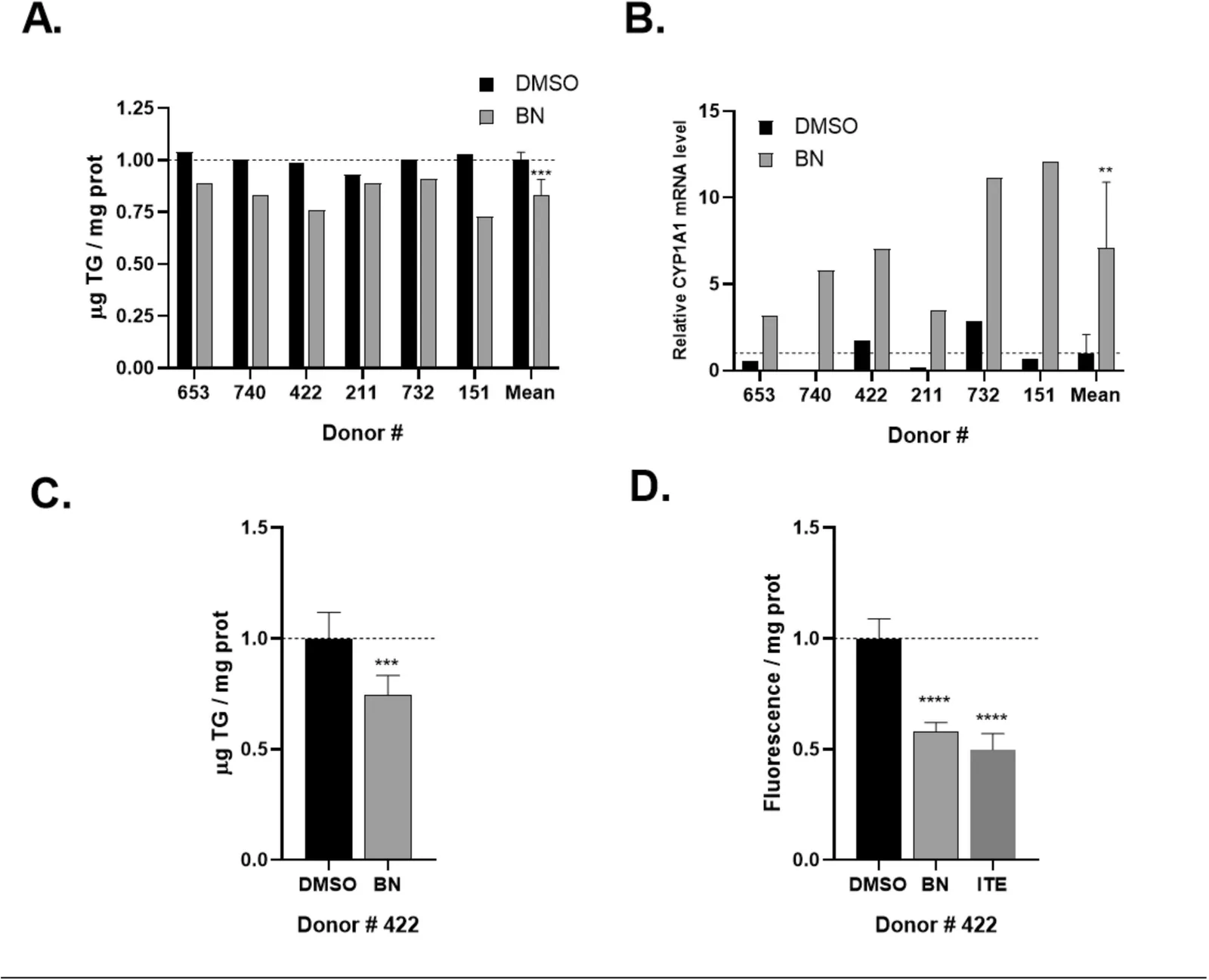
AHR activation reduces lipid accumulation in human hepatocytes. Hepatocytes were treated for 24 h with a fatty acid mixture (oleate:palmitate, 2:1, 0.6 mM) and DMSO, BN, or ITE. BN consistently reduced TG lipid content (**A**) and induced CYP1A1 mRNA expression across donors (**B**). In donor #422 (n >  = 5), both BN and ITE decreased intracellular TG levels measured by biochemical reaction (**C**) or Adipored fluorescent probe (**D**), supporting an anti-steatotic role of AHR. Averaged data are presented as mean ± SD and expressed as fold change with respect to DMSO treated cells (***, ****, *p* < 0.001 and *p* < 0.0001, respectively).

Intracellular neutral lipids were analysed using a TG extraction and quantification kit and the fluorometric probe AdipoRed in living cells. An initial screening was conducted across hepatocytes from multiple donors to assess TG accumulation and CYP1A1 expression levels. BN treatment consistently led to a reduction in lipid accumulation in all donors (Fig. 3A), accompanied by robust induction of CYP1A1 (Fig. 3B), the canonical AHR target.

Following the initial screening, a more detailed analysis was performed on hepatocytes from a donor with a robust response (#422) using both BN and ITE. Treatment with either agonist effectively reduced intracellular fat content (Fig. 3C-D) irrespective of the detection method, confirming an anti-steatotic role of AHR in human hepatocytes and supporting the notion that AHR downregulation in MASLD livers may contribute to disease progression.

### IGFBP1 identified as an AHR target gene with a potential anti-steatotic effect

Next, we performed whole microarray expression analyses of AHR regulated genes. Human hepatocytes were treated with 25 µM BN or DMSO for 24 h (n = 4) and whole transcriptomic analysis was performed to disclose gene expression changes triggered by AHR-dependent pathways.

Differential expression analysis was conducted using a filtering threshold of *p* < 0.05 and a fold change >  + 1.6 or < -1.6. Under these criteria, a total of 353 genes were significantly deregulated in response to BN treatment, of which 207 were upregulated and 146 were downregulated compared with DMSO-treated controls (Supplementary Table 4).

**Fig. 4:**
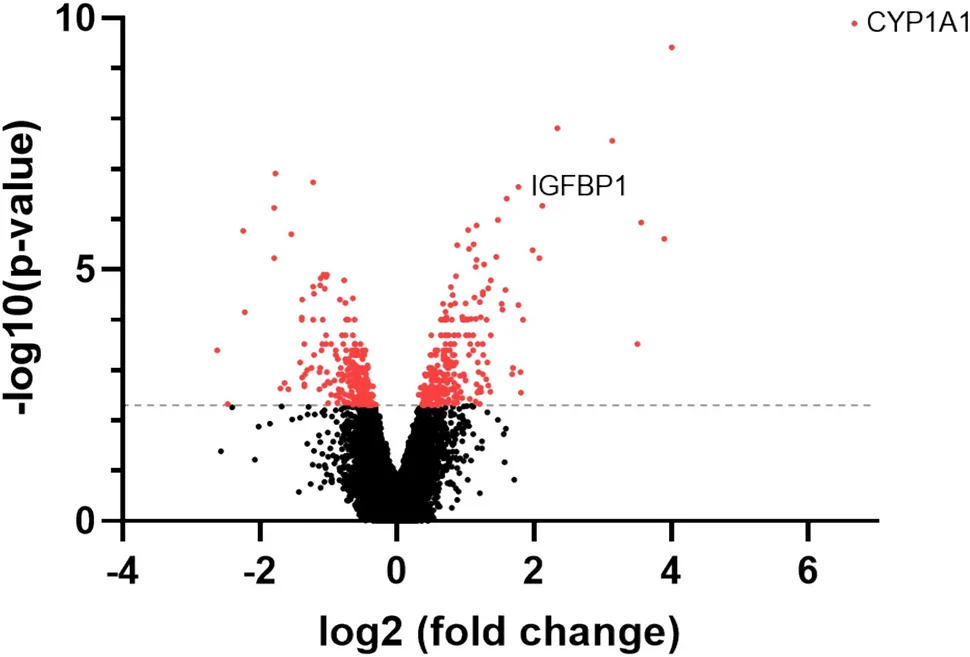
Volcano plot depicting differentially expressed genes in obese Upcyte cells treated with BN (n = 4) compared with cells treated with DMSO (n = 4). Each dot represents one gene, displayed according to its log2 fold change (x-axis) and -log10 (*p*-value) (y-axis). Significant upregulated and downregulated genes (± 1.6-fold change, *p* < 0.05) are highlighted in red.

The top 25 upregulated and downregulated genes are shown in Table 4, and the corresponding volcano plot is depicted in Fig. 4. The fold changes, calculated as the ratios of gene expression between BN-treated and vehicle-treated cells, ranged from + 102.08 (CYP1A1) to -6.13 (CER1).

**Table 4:** Top 25 upregulated and downregulated genes in BN versus DMSO treated hepatocytes as ranked by fold-change.

Gene	Fold change	*P*-value	FDR *P*-value	Description
CYP1A1	102,08	1,26E-10	2,70E-06	Cytochrome P450, family 1, subfamily A, polypeptide 1
NQO1	16,19	3,83E-10	4,11E-06	NAD(P)H dehydrogenase, quinone 1
AKR1B15	14,96	2,42E-06	0,0031	Aldo–keto reductase family 1, member B15
AKR1B10	11,8	1,15E-06	0,0021	Aldo–keto reductase family 1, member B10 (aldose reductase)
CIDEC	11,39	0,0003	0,0570	Cell death-inducing DFFA-like effector c
CLDN2	8,8	2,72E-08	0,0001	Claudin 2
EREG	5,04	1,54E-08	0,0001	Epiregulin
CYP1B1	4,35	5,40E-07	0,0013	Cytochrome P450, family 1, subfamily B, polypeptide 1
MYOF	4,23	5,87E-06	0,0053	Myoferlin
AREG	3,95	4,12E-06	0,0042	Amphiregulin
HAVCR1	3,58	0,0001	0,0307	Hepatitis A virus cellular receptor 1
CA9	3,5	0,0028	0,1739	Carbonic anhydrase IX
PLAU	3,48	0,0011	0,1124	Plasminogen activator, urokinase
AGR2	3,41	5,07E-05	0,0194	Anterior gradient 2, protein disulphide isomerase family member
IGFBP1	3,41	2,28E-07	0,0007	Insulin like growth factor binding protein 1
TFF1	3,24	0,0009	0,0996	Trefoil factor 1
ITGA2	3,21	0,0012	0,1150	Integrin, alpha 2 (CD49B, alpha 2 subunit of VLA-2 receptor)
STC2	3,03	3,84E-07	0,0010	Stanniocalcin 2
AKAP12	3,03	0,0144	0,3352	A kinase (PRKA) anchor protein 12
SLC22A18	2,99	2,53E-05	0,0136	Solute carrier family 22, member 18
EPHB6	2,95	0,0187	0,3776	EPH receptor B6
GRIA3	2,9	6,29E-05	0,0237	Glutamate receptor, ionotropic, AMPA 3
PIR	2,89	4,77E-05	0,0191	Pirin
NDRG1	2,78	0,0097	0,2924	N-myc downstream regulated 1
SPRY4	2,76	1,03E-06	0,0020	Sprouty RTK signaling antagonist 4
PPP1R1A	-2,55	0,0003	0,0527	Protein phosphatase 1, regulatory (inhibitor) subunit 1A
VCAM1	-2,57	0,0021	0,1579	Vascular cell adhesion molecule 1
CXCL10	-2,6	0,0014	0,1256	Chemokine (C-X-C motif) ligand 10
GLP2R	-2,61	3,93E-05	0,0176	Glucagon-like peptide 2 receptor
DEFB1	-2,61	9,87E-05	0,0307	Defensin, beta 1
ADAM22	-2,63	9,06E-05	0,0307	ADAM metallopeptidase domain 22
FREM1	-2,66	0,0007	0,0907	FRAS1 related extracellular matrix 1
PAH	-2,66	0,0088	0,2788	Phenylalanine hydroxylase
TSPAN2	-2,89	0,0095	0,2882	Tetraspanin 2
SPINK1	-2,92	1,97E-06	0,0026	Serine peptidase inhibitor, Kazal type 1
THBS1	-2,99	0,0024	0,1649	Thrombospondin 1
CYP3A4	-3,13	0,0018	0,1436	Cytochrome P450, family 3, subfamily A, polypeptide 4
HPX	-3,2	0,0053	0,2311	Hemopexin
LYPD6B	-3,25	0,0023	0,1617	LY6/PLAUR domain containing 6B
SCTR	-3,4	1,23E-07	0,0005	Secretin receptor
CFAP221	-3,45	5,94E-06	0,0053	Cilia and flagella associated protein 221
BCO2; RPS12P21	-3,46	5,93E-07	0,0013	Beta-carotene oxygenase 2; ribosomal protein S12 pseudogene 21
FLRT2	-3,59	0,0115	0,3097	Fibronectin leucine rich transmembrane protein 2
PTCHD4	-4,06	0,0133	0,3258	Patched domain containing 4
CYP3A7; CYP3A7-CYP3A51P	-4,67	6,96E-05	0,0253	Cytochrome P450, family 3, subfamily A, polypeptide 7; CYP3A7-CYP3A51P readthrough
UBD	-4,72	1,69E-06	0,0024	Ubiquitin D
SERPINA7	-5,25	0,0055	0,2313	Serpin peptidase inhibitor, clade A (alpha-1 antiproteinase, antitrypsin), member 7
AKR1D1	-5,55	0,0047	0,2211	Aldo–keto reductase family 1, member D1
KNG1	-5,95	0,0408	0,4926	Kininogen 1
CER1	-6,13	0,0004	0,0667	Cerberus 1, DAN family BMP antagonist

Functional pathway enrichment analysis revealed a total of 157 significantly enriched pathways associated with BN exposure (*p* < 0.01; Sources: Reactome, Wiki pathways and KEGG) (Supplementary Table 3B).

The most significantly upregulated pathways were primarily associated with glucose metabolism and metabolic adaptation. These included the Cori cycle, glycolysis and gluconeogenesis, and the pentose phosphate pathway, indicating a shift toward enhanced central carbon metabolism. In addition, HIF-1 signaling and the NRF2 pathway were markedly enriched, supporting boosting of hypoxia, antioxidant and detoxification defenses. Regulatory pathways such as the AHR pathway, nuclear receptors metapathway and estrogen metabolism were also significantly upregulated, pointing to the crosstalk of AHR with other nuclear receptors.

The most significantly downregulated pathways were predominantly associated with inflammatory signaling processes. These included the TNF signaling pathway, NF-kappa B signaling pathway, IL-17 signaling pathway, and chemokine receptors bind chemokines, all of which play central roles in cytokine production, immune cell recruitment, and amplification of inflammatory responses. Notably, their reduced expression following BN treatment and concomitant AHR activation suggests a dampening of pro-inflammatory signaling cascades. This anti-inflammatory signature may represent a beneficial effect of BN, aligning with the protective anti-steatotic role of AHR and supporting the translational relevance of these findings, as they are consistent with improved inflammatory profiles previously observed in human patients.

**Fig. 5:**
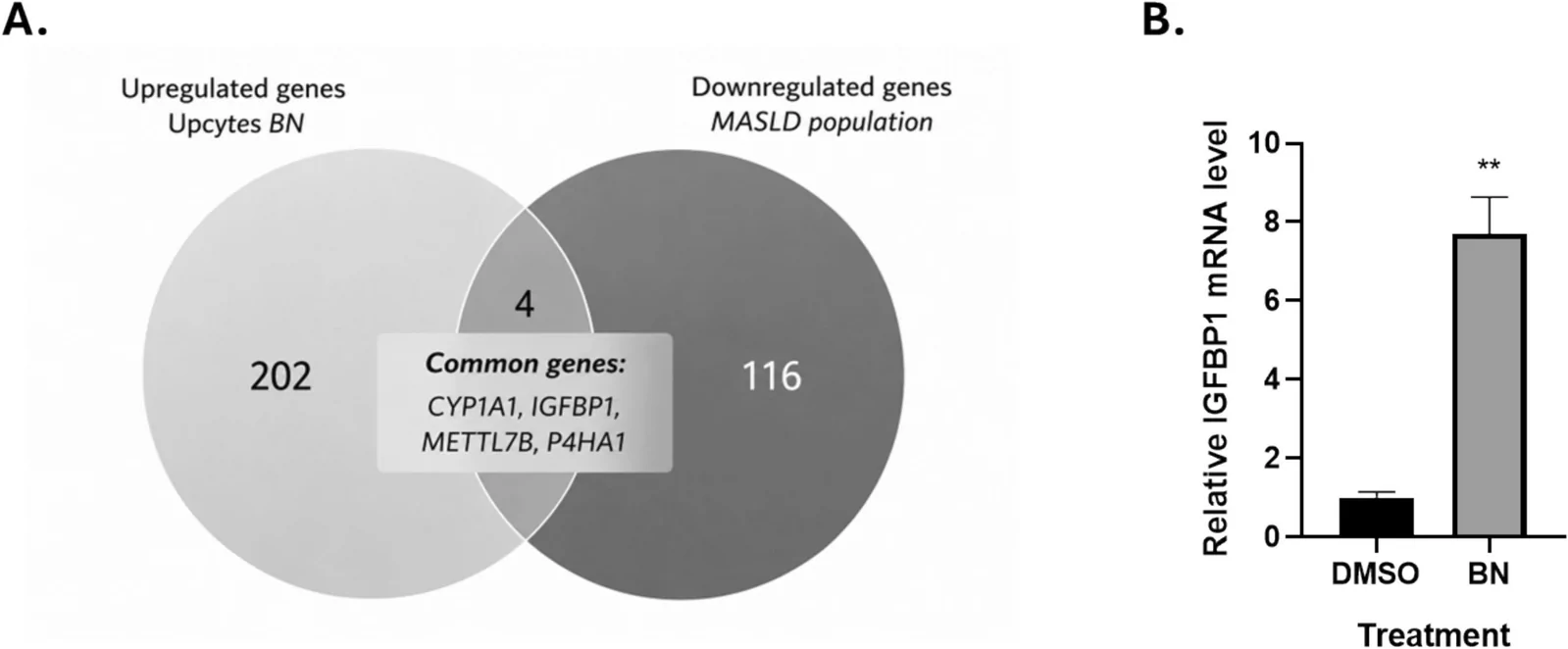
IGFBP1 identified as an AHR target gene with a potential anti-steatotic effect **A** Venn diagram of shared and unique genes between datasets. Venn diagram showing the overlap between unique genes upregulated in BN-treated Upcyte hepatocytes and genes downregulated in liver samples from MASLD patients. Four genes were common to both datasets: CYP1A1, IGFBP1, METTL7B, and P4HA1. **B** IGFBP1 mRNA levels. Cultured human hepatocytes were treated with 25 µM BN for 24 h and expression levels measured by RTqPCR. Data are presented as mean ± SD (**, *p* < 0.01).

A Venn diagram was used to identify overlapping genes between those upregulated in BN-treated hepatocytes and those downregulated in the obese MASLD patients. This analysis yielded four candidate genes: CYP1A1, IGFBP1, METTL7B, and P4HA1 (Fig. 5A).

Among the genes shared between datasets, we prioritized IGFBP1, given that the IGF1–IGFBP1 axis has been involved in metabolic liver diseases, including NAFLD/MASLD. RT-qPCR analysis confirmed a marked induction of IGFBP1 following AHR activation (Fig. 5B), supporting a potential link between AHR signaling and the regulation of hepatic glucose and lipid metabolism.

**Fig. 6:**
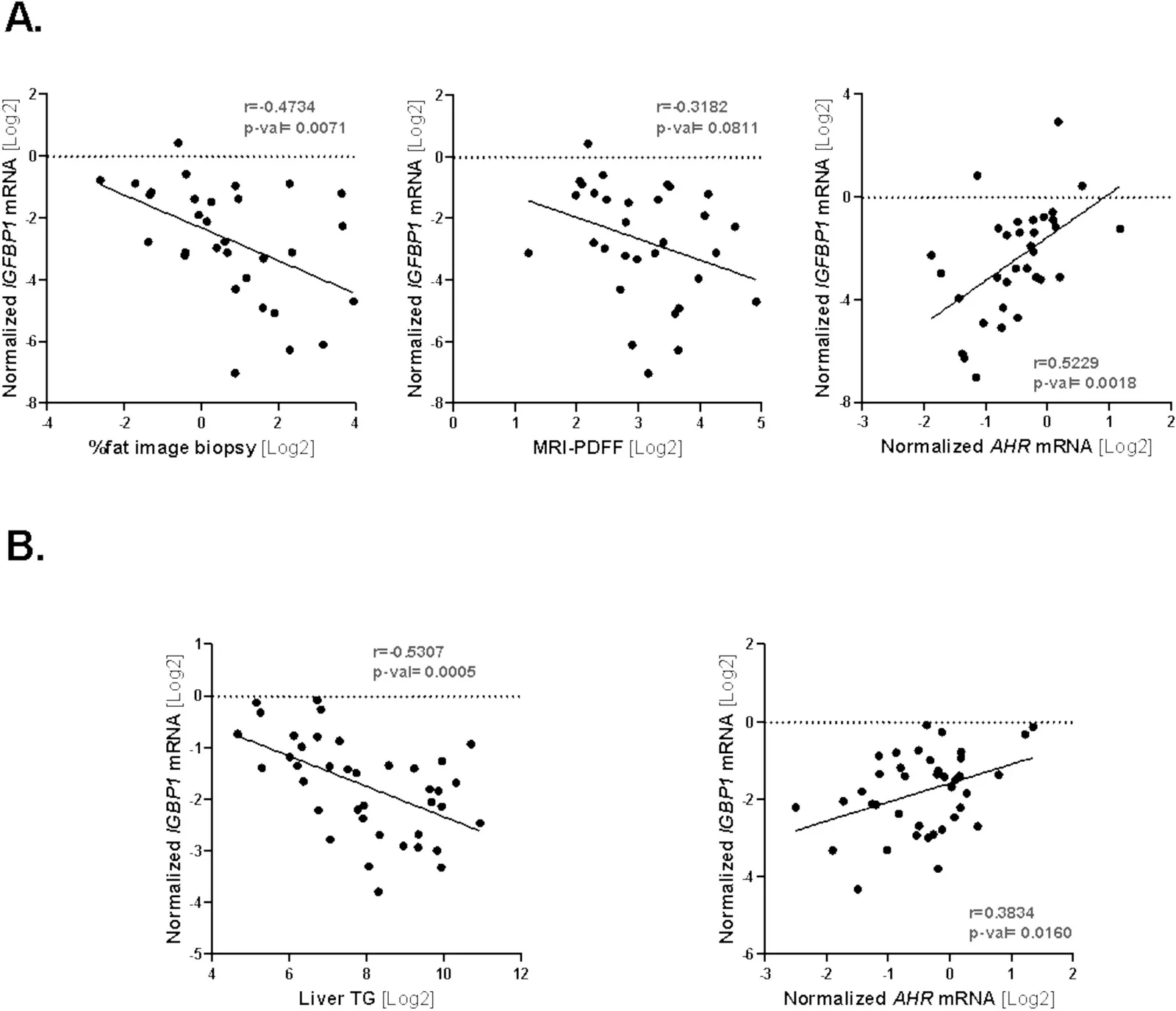
Correlation analyses of IGFBP1 expression levels with hepatic steatosis parameters. **A** Inverse correlations of IGFBP1 mRNA with % of liver fat and MRI-PDFF values, in obese patients undergoing bariatric surgery. A significant positive correlation between AHR and IGFBP1 expression is also depicted (right). **B** Associations in the non-obese liver biobank cohort, demonstrating negative correlations between IGFBP1 expression and hepatic TG levels, as well as a positive correlation between IGFBP1 and AHR expressions (right). r denotes Pearson’s correlation coefficient, measuring the strength and direction of the linear relationship between variables. The p-value (*p*-val) indicates the statistical significance of the correlation, testing whether the observed association differs from zero.

Consistent with this observation, IGFBP1 expression in human liver samples paralleled the pattern observed for AHR and its target gene CYP1A1. In obese patients undergoing bariatric surgery, hepatic IGFBP1 expression was inversely associated with liver fat content, as assessed by both digital biopsy imaging and MRI-PDFF, and positively correlated with hepatic AHR mRNA expression levels (Fig. 6A). These findings were further validated in the independent liver biobank cohort of non-obese patients (Fig. 6B).

Taken together, these results suggest that the AHR–IGFBP1 axis may contribute to the effects of AHR on hepatic lipid metabolism, providing a potential basis for its role in regulating TG accumulation and its relevance to MASLD pathophysiology.

### Anti-steatotic activity of IGFBP and IGF1 in cultured human hepatocytes

Based on our initial observations linking AHR activation to increased IGFBP1 expression, we next investigated whether IGFBP1 and its ligand, IGF1, could contribute to the reduction of hepatocellular lipid content observed upon AHR activation. To address this, human Upcyte hepatocytes were treated with recombinant IGFBP1 and IGF1, either alone or in combination, and intracellular lipid accumulation was quantified using the AdipoRed probe.

**Fig. 7:**
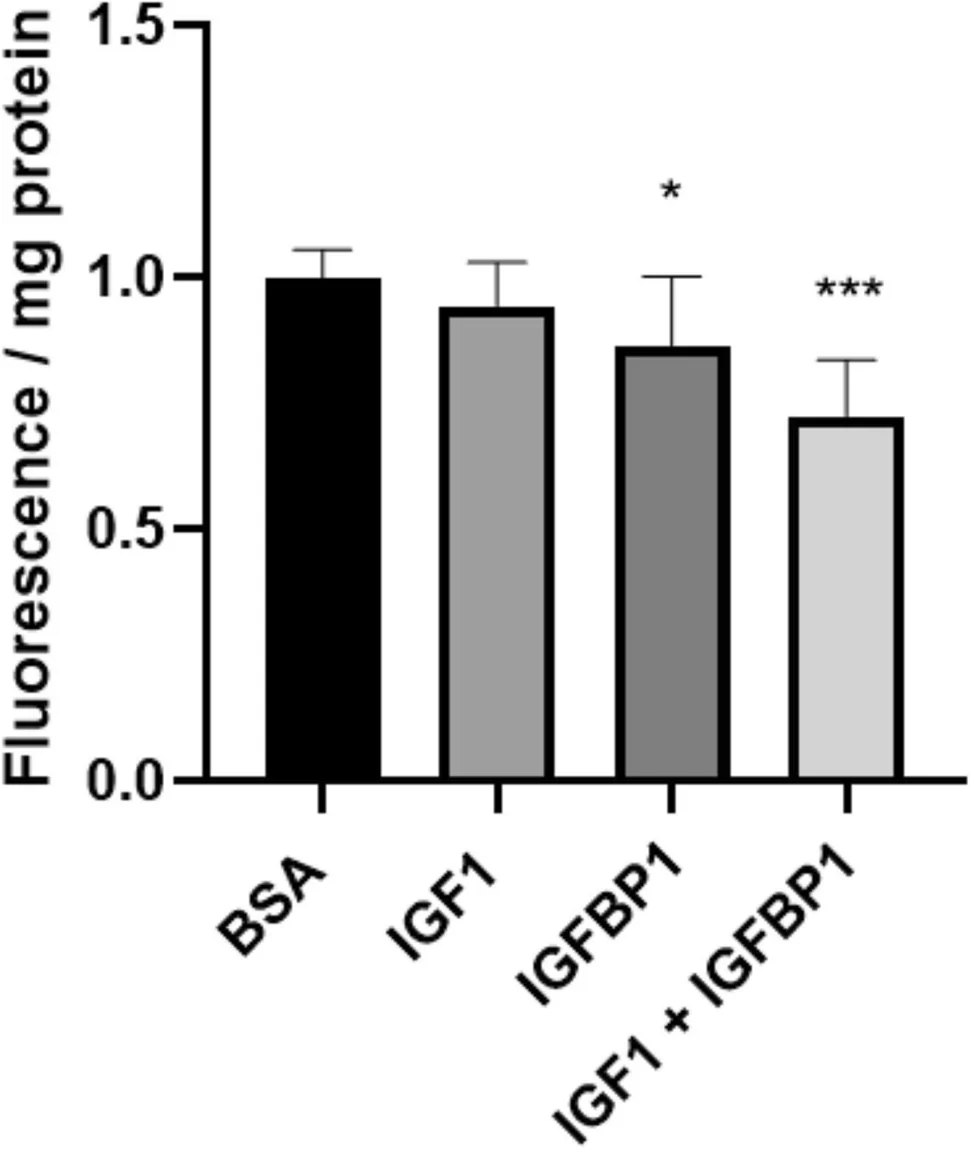
Effects of IGF1 and IGFBP1 on intracellular lipid accumulation in human Upcyte hepatocytes. Human Upcyte hepatocytes were treated with recombinant IGF1, IGFBP1, or IGF1 + IGFBP1, and intracellular lipid accumulation was quantified using the AdipoRed probe. Fluorescence values were normalized to total protein content. Data are shown as mean ± SD (n = 6) and are expressed as fold-change with respect to solvent-treated cells (BSA) (*, ***, *p*-value < 0.05 and 0.001, respectively).

As shown in Fig. 7, treatment with IGFBP1 significantly reduced intracellular lipid accumulation compared with control conditions (BSA), supporting a direct anti-steatotic effect in human hepatocytes. IGF1 alone produced a non-significant slight decrease, whereas the combined treatment with IGF1 and IGFBP1 resulted in the largest reduction in lipid content, suggesting an additive or cooperative effect between both factors. These findings indicate that IGFBP1 is not only induced downstream of AHR activation, but may also act as an effector of its metabolic response by limiting TG accumulation in hepatocytes.

Taken together, these results support the hypothesis that the AHR–IGFBP1 axis exerts a protective, anti-steatotic role in hepatocytes and its repression may contribute to lipid accumulation in the context of MASLD.

## Discussion and conclusions

To our knowledge, hepatic AHR expression has not been systematically evaluated in steatosis/MASLD patient cohorts with a quantitative characterization of liver fat. In this study, we show that hepatic AHR expression, together with the canonical AHR target gene CYP1A1, is inversely associated with liver fat content in two independent cohorts. In parallel, activation of AHR in fatty acid-loaded human hepatocytes reduced intracellular lipid accumulation, and transcriptomic analyses identified IGFBP1 as an AHR-inducible gene that was also downregulated in steatotic human liver. Consistently, recombinant IGFBP1 reduced lipid accumulation in cultured human hepatocytes. Taken together, these findings support the existence of an AHR-associated anti-steatotic program in the human liver and point to IGFBP1 as a plausible downstream mediator of this response.

The observed repression of the AHR pathway in steatotic human liver does not, by itself, indicate whether reduced AHR signaling is pathogenic or adaptive. This issue has remained controversial in the literature, largely because experimental studies have reported apparently opposite outcomes depending on the model used (Patil et al. 2023). In multiple mouse models, constitutive or sustained AHR activation by high-affinity xenobiotic ligands such as TCDD has been associated with steatosis, dyslipidaemia, oxidative stress and impaired mitochondrial function. Conversely, AHR deletion or pharmacological antagonism has been reported to protect against HFD-induced steatosis in some settings (Patil et al. 2023), whereas in others, including hepatocyte-specific Ahr deficiency, the absence of AHR aggravated steatosis and liver injury (Wada et al. 2016). Altogether, these observations support the idea that the metabolic outcome of AHR signaling is highly context dependent and may be determined by ligand identity, affinity, duration of exposure, tissue specificity, and metabolic background.

In addition, emerging evidence implicates endogenous and microbiota derived AHR ligands (indole derivatives, sulforaphane, and tryptophan catabolite cinnabarinic acid) as hepatoprotective, reducing lipogenesis, oxidative stress and inflammation, and improving metabolic homeostasis in experimental NAFLD/MASH models (Patil et al. 2025). Other agonists such as BN have a much shorter half-life than the prototypical persistent AHR agonists (e.g. TCDD and related dioxins). BN is mainly metabolized by CYP1A enzymes, which AHR potently induces, leading to rapid oxidative biotransformation and clearance (Vyas et al. 1983). Thus, differences in AHR activation times may also explain the different effects observed with sustained, toxic AHR activation vs transient hepatoprotective activation (Oesch-Bartlomowicz et al. 2005).

Our results in cultured human hepatocytes favor an anti-steatotic role of AHR under conditions of transient activation. This interpretation is consistent with previous work showing that activation of AHR by BN reduced the expression of fatty acid synthesis genes such as FASN, ACACA and SCD1 in human hepatocytes, although net intracellular lipid accumulation was not directly measured (Tanos et al. 2012). By contrast, primary human hepatocytes exposed to TCDD accumulated fatty acids despite showing a transient repression of lipogenic genes, including FASN and ACLY, suggesting that the final metabolic outcome of AHR activation depends on the balance among fatty acid uptake, synthesis, oxidation and secretion (Tanos et al. 2012). One possible explanation is that persistent toxic agonists and transient agonists do not trigger exactly the same programs.

A relevant downstream effector repeatedly implicated in AHR-driven steatosis is the fatty acid transporter CD36. Treatment of primary human hepatocytes or Huh-7 cells with AHR agonists (not BN) has been reported to induce CD36 expression (Lee et al. 2010), and several mouse studies have also highlighted CD36 as a key AHR-regulated driver of hepatic lipid uptake. However, our data did not support a prominent role for this axis in the human samples analyzed here, since we did not observe a correlation between hepatic AHR and CD36 mRNA levels in human liver samples, nor did we detect induction of CD36 after BN treatment in human hepatocytes (data not shown). Although these negative findings do not exclude a role for CD36 under other conditions or with other ligands, they suggest that the anti-steatotic effects of AHR activation in human hepatocytes may involve downstream programs different from the CD36-centered mechanisms described in some models of persistent xenobiotic exposure.

Our results also suggest that caution is warranted when interpreting the current AOP of toxic steatosis, where it is assumed that AHR activation is always pro-steatotic, with, among others, the activation of downstream Key Events such as CD36 (Mellor et al. 2016).

An important finding of the present study is the identification of IGFBP1 as one of the few genes that were upregulated by AHR activation in hepatocytes and, simultaneously, positively correlated with AHR expression in the human liver, where it shows inverse association with steatosis. As the IGF-IGFBP1 axis has been implicated in metabolic liver diseases (Clemmons 2012), it is tempting to postulate IGFBP1 as a downstream anti-steatotic effector of the AHR pathway.

Regarding the negative correlation of IGFBP1 mRNA with liver steatosis, our results are in agreement with previous studies showing that serum fasting IGFBP1, and its phosphorylated form, inversely correlate with liver fat (Minami et al. 2008; Petäjä et al. 2016). Since the liver is a major source of circulating IGFBP1, our results support that the decrease in serum IGFBP1 in NAFLD comes from lower synthesis in hepatocytes, likely because of reduced AHR levels.

Previous studies are consistent with the view that IGFBP1 is an AHR-responsive gene in hepatocytes. In mice, hepatic IGFBP1 mRNA levels were reported to be higher in AHR knockout animals than in wild-type mice, yet TCDD induced IGFBP1 in an AHR-dependent manner in wild-type but not in AHR-null mice, indicating a complex relationship between basal and inducible AHR signaling (Minami et al. 2008). In addition, a xenobiotic responsive element has been identified in the promoter region of the IGFBP1 gene, and TCDD has been shown to induce IGFBP1 in human hepatocytes and HepG2 cells (Marchand et al. 2005). Taken together, the published evidence and our present results support the notion that IGFBP1 is an AHR-responsive gene in hepatocytes and that its expression is reduced in steatotic human liver.

The biological relevance of this observation is reinforced by our functional data showing that recombinant IGFBP1 reduced lipid accumulation in cultured human hepatocytes. These findings are in line with previous work showing that IGFBP1 attenuates fatty acid-induced lipid accumulation in hepatocytes and ameliorates steatosis and liver injury in experimental models (Pan et al. 2021). Mechanistically, that study linked IGFBP1 to reduced expression of lipogenic regulators such as SREBP-1c, FASN and SCD1, increased expression of β-oxidation-related genes including PPARα and CPT1, and attenuation of inflammatory signaling through the NF-κB/ERK pathway (Pan et al. 2021). Although these mechanisms were not directly addressed in our study, they provide a plausible framework through which AHR-induced IGFBP1 may contribute to limiting hepatocellular lipid accumulation.

Our data suggest that IGF1 may also modulate this response. Although IGF1 alone produced only a modest effect in our in vitro model, the combination of IGF1 and IGFBP1 yielded the greatest reduction in lipid accumulation. This observation is compatible with previous studies indicating that IGF1 is reduced in individuals with more severe steatosis and higher NAFLD activity score (Stanley et al. 2020), and that IGF1 can regulate hepatic inflammation and lipid metabolism through IGF1R–PI3K–Akt signaling (Gui et al. 2023). IGFBP1 can modulate cellular responses by regulating the bioavailability and cellular actions of IGFs, but also independently of IGF/IGF receptors via integrin-binding motifs (Haywood et al. 2016). Nevertheless, our results do not allow firm conclusions regarding the functional interaction between IGF1 and IGFBP1, and this point will require further mechanistic investigation.

Several limitations of this study should be acknowledged. First, the associations identified in human liver tissue do not establish causality. Second, AHR activity in the patient cohorts was inferred mainly from CYP1A1 expression, and direct assessment of receptor binding or promoter occupancy was not performed. Third, we did not directly manipulate AHR in hepatocytes using loss-of-function or overexpression approaches. Although BN and ITE are well-demonstrated AHR agonists, the absence of genetic validation prevents us from concluding that these effects are exclusively AHR-dependent. Fourth, while IGFBP1 emerged as a strong candidate mediator of the anti-steatotic effect of AHR, other potential downstream mechanisms have not been ruled out.

Despite these limitations, the present study provides translational evidence that is directly relevant to human MASLD. The translational design of our study combines quantitative analyses in the human liver with mechanistic observations in cultured human hepatocytes. The results strongly support a protective association between AHR signaling and hepatic steatosis in humans. Even though further studies will be required to firmly establish causality, these findings narrow an important gap between conflicting previous studies and human disease, and identify the AHR–IGFBP1 axis as a promising framework for future mechanistic and therapeutic studies in MASLD.

## Supplementary Information

Below is the link to the electronic supplementary material.

Supplementary file1 (XLSX 11 KB)

Supplementary file2 (XLSX 90 KB)

Supplementary file3 (XLSX 35 KB)

Supplementary file4 (XLSX 37 KB)

## Data Availability

Microarray data have been deposited in the Zenodo repository under https://doi.org/10.5281/zenodo.20079491
